# Use of Reticulated Hyaluronic Acid Alone or Associated With Ozone Gas in the Treatment of Osteoarthritis Due to Hip Dysplasia in Dogs

**DOI:** 10.3389/fvets.2020.00265

**Published:** 2020-05-13

**Authors:** José I. S. Silva Júnior, Sheila C. Rahal, Ivan F. C. Santos, David J. C. Martins, Fernanda Michelon, Maria J. Mamprim, Rubia M. Tomacheuski, Luiz E. C. S. Correia

**Affiliations:** ^1^Department of Veterinary Surgery and Animal Reproduction, School of Veterinary Medicine and Animal Science, São Paulo State University (UNESP), Botucatu, Brazil; ^2^Department of Animal Science, School of Agricultural and Veterinary Studies, UNESP, Jaboticabal, Brazil

**Keywords:** viscosupplementation, articular degeneration, pain, treatment, dysplasia

## Abstract

This study aimed to evaluate reticulated hyaluronic acid alone or associated with ozone gas in the treatment of osteoarthritis due to hip dysplasia in dogs. Fourteen client-owned dogs were randomly assigned into two groups: Group 1—single intra-articular injection of hyaluronic acid; Group 2—single intra-articular infiltration injection of hyaluronic acid associated with ozone gas. Each hip joint received an average of 0.75 mL of reticulated hyaluronic acid ultrasound-guided. Ozone gas at a dose of 45 μg/mL was incorporated into hyaluronic acid by insufflation. Dogs were evaluated for body condition scoring, orthopedic examination and radiographic scores of the hip joints, goniometric measurements of the hip joints, visual gait score, and kinetic analysis. The evaluations were conducted immediately before treatments (M0), and at days 30 (M1), 60 (M2), and 90 (M3) after treatments. There were no significant differences in body mass and body condition scoring (5-point scale) in each group in all evaluation moments. The scores of orthopedic examination of the hip joints showed statistical differences in each group between moments (M0 > M3), but differences were not observed between groups. No statistical differences were found for radiographic scores in each group between moments, but differences were observed between groups immediately prior to treatments (G1 > G2) and 90 (G1 > G2) after treatments. Goniometric measurements of hip flexion and extension showed no significant differences in each group between moments or between groups. No statistical differences between groups were found concerning the lameness score. There were significant differences for lameness score among moments in Group 1, being M0 > M2 and M0 > M3, and Group 2 in which M0 > M1, M0 > M2, and M0 > M3. The mean percentage of change of PVF and VI between M3 and M0 in Group 1 was almost null and in Group 2 was positive, being 31.1 ± 29.4 and 10.6 ± 25.4, respectively. In conclusion, the intra-articular viscosupplementation alone or associated with ozone gas allowed improvement of lameness scores and orthopedic examination score. In Group 2 the association of ozone gas had better results on kinetic analysis.

## Introduction

Hip dysplasia is an orthopedic disease considered very frequent in dogs even with several control-breeding programmes (1). It is considered a biomechanical disease related to anomalous development of the hip joints that begins after birth and progresses during life, and after the establishment of fibrosis and osteoarthritis may show improvement of the stabilization (2, 3). The main goals of hip dysplasia treatment are to obtain relieve pain and to maintain an adequate gait and weight-bearing, as well as to decrease disease progression (1–4).

Adult dogs with hip joints already affected by osteoarthritis may receive surgical treatment (total hip replacement, hip denervation, or femoral head and neck excision) or non-surgical management (weight control, physiotherapy, environment changes, exercise restriction, medications, supplements, acupuncture, or regenerative medicine), being the choice determined by environmental factors and general healthy, dog's temperament, owner's financial conditions, and presence of comorbidities (2–7).

Some of the medications or supplements used are non-steroidal anti-inflammatory drugs, pain relief medication, antioxidants, and chondroprotective drugs (2, 4, 6). Another treatment modality still little explored in the treatment or prevention of naturally acquired osteoarthritis in dogs is viscosupplementation (8–10). Viscosupplementation refers the intra-articular administration of exogenous hyaluronic acid, or hyaluronic acid derivatives to provide pain relief and improve joint mobility (11–13).

Hyaluronic acid is classified as glycosaminoglycan whose molecules interconnect to make a high viscosity solution (12). In the normal joint, hyaluronic acid is responsible for providing synovial fluid viscosity and elasticity, but its concentration and molecular weight are reduced in joint with osteoarthritis (4, 11, 14).

The decreased viscoelasticity of the synovial fluid increases the susceptibility for the development of injuries due to cartilage overload (15). Some mechanisms have been related to the therapeutic effects of the hyaluronic acid, including anti-inflammatory and anti-nociceptive effects, reestablishment of elastic and viscous properties of the synovial fluid, normalization of synthesis of hyaluronan by synoviocytes (11, 14).

In turn, intra-articular ozone has been used in human patients with osteoarthritis to reduce pain, relieve of physical disability and stiffness, in order to promote the reduction of joint inflammation and to improve quality of life (16–21). The action mechanism is not fully understood (20), but a hypothesis it that ozone injected into synovial fluid produces form reactive oxygen species and lipid oxidative products (17). Thus, the ozone in osteoarthritis may be responsible for cell metabolism activation, to reduce prostaglandin synthesis and oxidative stress, and to induce antioxidant enzyme synthesis, as well as to augment oxygen supply to tissues, promoting immunomodulatory effect and improving vascularization, among others (17, 20).

Very few studies have investigated the role of viscosupplementation and ozone gas in canine hip dysplasia (10). Therefore, this study aimed to evaluate reticulated hyaluronic acid alone or associated with ozone gas in the treatment of osteoarthritis due to hip dysplasia in dogs. The hypothesis was that gas ozone inclusion induces a better clinical outcome compared to hyaluronic acid alone.

## Materials and Methods

### Dog Selection

This study was approved by the Institutional Ethics Committee for the Use of Animals (n°. 0101/2018—CEUA). A written informed consent form was signed from each dog's owner before the initiation of the study.

Twenty-three adult dogs diagnosed with osteoarthritis due to hip dysplasia were evaluated. Dogs were selected based on clinical signs; general physical examination, orthopedic and neurologic exams; hematological and serum biochemical tests, including for alanine aminotransferase, urea and creatinine; and radiographic evaluation of the hip joints. The inclusion criteria were hip dysplasia dogs exhibiting clinical signs of pain and lameness, and without any history of previous surgery. The exclusion criteria were dogs submitted to any other surgical procedure in the previous 6 months before the study, dogs receiving anti-inflammatory drugs, presence of other musculoskeletal, and/or neurological conditions.

### Treatments

The dogs were randomly assigned into two groups: Group 1—single intra-articular injection of hyaluronic acid; Group 2—single intra-articular infiltration injection of hyaluronic acid associated with ozone gas. Each hip joint received an average of 0.75 mL of hyaluronic acid alone^1^ (8 mg/grams) or associated with ozone gas. Ozone gas at concentration of 45 μg/mL was incorporated into hyaluronic acid by insufflation using sterile hypodermic needle 21G ×1 1/2″ (0.8 ×40 mm). Ozone was provided by model 0 & L3.0 RM ozone generator^2^.

After general anesthesia *(RMT)* with propofol, each dog was positioned in lateral recumbency to perform ultrasound-guided^3^ *(FM)* intra-articular injection in the right and left hip joints. In the area of injection, the hair was clipped and site was disinfected with chlorhexidine. The intra-articular injection was done *(JISSJ)* with needle (21G ×1 1/2″) or mandrel of the 20G catheter attached to 1 mL syringe inserted at the midpoint of the proximal edge of the greater trochanter.

### Body Condition Scoring (BCS)

A 5*-*point scale was used to evaluate BCS (22), which were conducted *(JISSJ)* immediately prior to treatments (M0), and at days 30 (M1), 60 (M2) and 90 (M3) after treatments.

### Hip Examination and Radiographic Evaluation of the Hip Joints

The scores of orthopedic examination of the hip joints *(JISSJ)* based on signs of crepitation and pain on palpation were: 1 - absent, 2 - mild, 3 - moderate, 4 - severe.

Ventrodorsal hip-extended radiographs were performed *(MJM–FM)* under general anesthesia. After 8-h fast, the dogs received pre-medication with acepromazine (0.05 mg/kg) and morphine sulfate (0.5 mg/kg) intramuscularly, followed by anesthetic induction and maintenance with propofol (5 mg/kg, IV). Digital radiographs^4^ were done with a 1 m focus-film distance, 60–90 kV, and 5.0–6.4 mAs.

Scoring radiographs for hip dysplasia were based on the Orthopedic Foundation for Animals (OFA) classification (23): 0–normal hip (excellent, good and fair classification), 1–borderline, 2, mild, 3–moderate, and 4–severe. Norberg angle was measured for each hip using a commercial software^5^, as previously described (24). All images were stored in Synapse PACS system (Fujifilm) as DICOM-formatted files.

The evaluations were performed immediately prior to treatments (M0) and at day 90 (M3) after treatment.

### Goniometric Measurements

The goniometric measurements of the hip joints *(JISSJ)* were carried out using plastic universal goniometer^6^, as previously described (25). The dogs were positioned in lateral recumbency and one arm of the goniometer was placed on the axis longitudinal of the femur (greater trochanter to lateral femoral epicondyle of the femur) and other arm on the line sacral tuberosity of the ilium to the ischial tuberosity. Hip joint flexion and extension were determined. The measurements were carried out in triplicate by the same investigator immediately prior to treatments (M0) and at day 90 (M3) after treatments and was selected the median value for statistical analysis.

### Lameness Evaluation

Lameness at walk was evaluated (JISSJ) using a visual gait score, based on previously reported (26): 0 (normal use of the limb), 1 (lameness is intermittent), 2 (lameness is evident, but dog shows weight-bearing), 3 (lameness is severe, but dog shows weight-bearing), 4 (intermittent lameness, but the dog did not shows weight-bearing), 5 (the limb is not used). All dogs were filmed during gait analysis.

The evaluations were conducted immediately prior to treatments (M0), and at days 30 (M1), 60 (M2) and 90 (M3) after treatments.

### Kinetic Gait Analysis

After acclimatization and familiarization with the environment and pressure-sensitive walkway, each dog was guided to the right of the handler to walk *(JISSJ)* (velocity 0.9–1.1 m/s, acceleration −0.2–0.2 m/s^2^) in a straight line over the pressure*-*sensitive walkway^7^. The system was calibrated as specified by the manufacturer. Approximately 15 trials were obtained for each dog and the first five valid trials were used. Valid trials included those that all four limbs had contact on surface of the pressure-sensitive walkway with the dog maintaining the head in an adequate position during walking. The acquisition and analysis of the data were done using a specific software^8^. The Peak Vertical Force (PVF) and Vertical Impulse (VI) were normalized according to dog's body weight and represented as a percentage of body weight (%BW). The percentage change of the PVF (%BW) and the VI (%BW × s) were calculated as previously described (27): $\frac{\left[\left(X2\right)-\left(X1\right)\right]}{\left(X1\right)}\times \;100$

The data were collected and analyzed immediately before treatments (M0) and at day 90 (M3) after treatments.

### Statistical Analysis

Categorical data of BCS, scores of orthopedic examination, scoring radiographs, and visual gait score were directly converted as treated as continuous variables for statically analysis *(LECSC)* (purposes (28, 29). The variables BCS and visual gait score were evaluated at dog level, and other variables such as scores of orthopedic examination, scoring radiographs, Norberg angle, goniometric measurements, and kinetic variables were evaluated at joint level. All the analyses were carried out using the statistical software SAS, version 9.3. After data were tested for Gaussian distribution using Shapiro-Wilk normality test, non-parametric tests were used; the Mann Whitney U to compare data between Groups 1 and 2, the Wilcoxon Signed Ranks to compare follow-up data of groups M0-M3, and the Kruskal-Wallis test followed by Dunn test to compare lameness data of M0-M1-M2-M3. A *P* < 0.05 was considered significant.

## Results

Of 23 dogs evaluated, 14 met the inclusion criteria that were randomly assigned to two groups. Group 1 (*n* = 7) was composed of four males and three females, three neutered, and four entire, average age of 5.9 ± 2.3 years, average body mass of 38.3 ± 13.8 kg, being three crossbreds, two German shepherds, and two Great Danes. Group 2 (*n* = 7) was composed of three males and four females, six neutered, and one entire, average age of 6.4 ± 2.7 years, average body mass of 33.6 ± 12.6 kg, being three German shepherds, two crossbreds, one Labrador retriever, and one Rottweiler. Dogs were numbered from 1 to 7 for Group 1, and 8–14 for Group 2.

In both groups, the dogs showed no signs of complications due to intra-articular injections. No statistical differences were found between groups and in each group among moments for body mass. BCS were not affected by the treatments. In Group 1, 71.43% (*n* = 5) of the dogs had score three and 28.57% (*n* = 2) had score 4. In Group 2, 42.85% had score 3 (*n* = 4) and 57.14% score 4 (*n* = 3).

### Scores of Orthopedic Examination and Radiographic Evaluation of the Hip Joints

The scores of orthopedic examination of the hip joints showed statistical differences in each group between moments (M0 > M3), but differences were not observed between groups (Table 1).

**Table 1:** Body condition scoring (BCS), orthopedic examination score (Orthop), hip radiograph score (X-ray), Norberg Angle (NA), hip extension, hip flexion, percentage change of Peak Vertical Force (PVF) and percentage change of Vertical Impulse (VI) of the hind limbs with osteoarthritis due hip dysplasia, immediately prior to treatment (M0) and at day 90 after (M3) intraarticular injection of hyaluronic acid (Group 1, 7 dogs, 14 hind limbs), or hyaluronic acid associated with ozone gas (Group 2, 7 dogs, 14 hind limbs).

**Variables**	**G1**					**G2**					**M-W test *P*-value**
	**N^**°**^**	**Min**	**Max**	**Mean ± SD**	**Mean rank**	**N^**°**^**	**Min**	**Max**	**Mean ± SD**	**Mean rank**	
BCS	7	3	4	3.29 ± 0.49	7.0	7	3	4	3.43 ± 0.53	8.0	0.32
Orthop score/M0	14	2	4	2.8 ± 0.7^a^	14.5	14	2	4	2.8 ± 0.7^a^	14.5	1
Orthop score/M3	14	2	3	2.4 ± 0.5^b^	14.5	14	2	3	2.4 ± 0.5^b^	14.5	1
X-ray score/M0	14	2	4	3.4 ± 0.8^a^	10.2	14	2	3	2.6 ± 0.5^a^	18.8	0.01
X-ray score/M3	14	2	4	3.4 ± 0.8^a^	10.2	14	2	3	2.6 ± 0.5^a^	18.8	0.01
NA degrees/M0	14	68	122	94.5 ± 19.0^a^	13.3	14	82	115	101.8 ± 9.5^a^	15.7	0.45
NA degrees/M3	14	68	117	93.9 ± 19.0^a^	13.3	14	80	114	101.1 ± 10.4^a^	15.7	0.45
Hip extension (degrees)/M0	14	76	112	96.4 ± 10.5^a^	12.3	14	66	128	103.1 ± 17.0^a^	16.7	0.16
Hip extension (degrees)/M3	14	68	115	97.4 ± 15.1^a^	12.3	14	66	118	103.9 ± 13.9^a^	16.8	0.15
Hip flexion (degrees)/M0	14	42	76	61.1 ± 10.4^a^	12.1	14	44	92	68.6 ± 14.6^a^	16.8	0.14
Hip flexion (degrees)/M0	14	48	74	59.9 ± 10.0^a^	11.6	14	46	94	72.1 ± 16.7^a^	17.4	0.06
PVF change M3-M0 (%)	14	−33.1	26.8	1.3 ± 19.9	10.6	14	−19.3	78.7	31.1 ± 29.4	18.4	0.01
VI change M3-M0 (%)	14	−66.7	32.1	−2.3 ± 25.5	12.6	14	−32.5	44.6	10.6 ± 25.4	16.4	0.25

*Key: %-Percentage*;

a, b * Variables with different superscripts letters are significantly different in M0 and M3 (P < 0.05, Wilcoxon Signed Ranks test); M-W test-Mann-Whitney U test; Max- Maximum; Min-Minimum; N°-Number*.

In Group 1, right and left hip joints of each dog had similar radiographic scores, before and after the treatment, being 57.14% had severe classification, 28.57% moderate, and 14.71% mild. In Group 2, right and left hip joints of each dog had similar radiographic scores, before and after the treatment, being 57.14% had moderate classification, and 42.85% mild. No statistical differences were found for radiographic scores in each group between moments, but differences were observed between groups immediately prior to treatments (G1 > G2) and 90 (G1 > G2) after treatments (Table 1). No statistical differences were found between groups and in each group between moments for Norberg angle (Table 1).

### Goniometric Measurements

Goniometric measurements of hip flexion and extension showed no significant differences in each group between moments or between groups (Table 1).

### Lameness Evaluation

No statistical differences between groups were found concerning the lameness score. There were significant differences for lameness score among moments in Group 1, being M0 > M2 and M0 > M3, and Group 2 in which M0 > M1, M0 > M2, and M0 > M3. There were no significant difference (*P* > 0.05) between moments M0-M1, M1-M2, M1-M3, and M2-M3 in Group, and between M1-M2, M1-M3, and M2-M3 in Group 2.

### Kinetic Analysis

The mean percentage of change of PVF in M3 was positive in both groups and in Group 2 was bigger than in Group 1 (*P* < 0.05). The mean change VI between M3 and M0 it was also positive in Group 2 and better than in Group 1 (Table 1).

## Discussion

The present study compared intra-articular injection of hyaluronic acid alone or associated with ozone gas in dogs with osteoarthritis due to hip dysplasia and did not observe better outcome in dogs that received the association, except for kinetic data.

In both groups, average body mass including standard deviation corresponded to medium to large size dogs; the German shepherd breed was the most represented (36%). In general, hip dysplasia is more prevalent in medium to large breeds, brachycephalic breed, and also dogs with a high proportion of body length to height (3, 7).

The Body Condition Scoring showed that 35.71% (*n* = 5) of the dogs were classified as overweight. The excess of body mass contribute to increase joint stress that may cause cartilage degradation (3). In addition, a correlation between obesity and decreased ability to perform exercise has been observed in dogs with hip dysplasia (30). On the other hand, no statistical differences were found in body mass of the dogs within each of the groups among evaluation moments, thus maintaining the uniformity of the sample. It should be considered that weight reduction alone could improve clinical lameness in overweight dogs with hip dysplasia (31).

In both groups, the right and left hip joints of each dog had similar radiographic scores. Radiographic changes in hip dysplasia often do not correspond to clinical presentation, and approximately 25% of dogs may also have spinal lesions (2, 7). Although there were no significant differences in lameness scores between groups, in the evaluation among moments in each group was observed improvement in lameness score after both treatments with intra-articular viscosupplementation. The hyaluronic acid used in the present study had non-avian origin. Hyaluronic acids produced by bacterial fermentation has lower allergenic potential in comparison those avian origin (11, 12). In the present study, no complications related to the intra-articular injection were observed in both groups. However, adverse effects such as arthralgia, effusion, and heat have been observed in some human patients after intra-articular hyaluronic acid injection in knee (11–13).

The hyaluronic acids have been classified in low molecular weight (0.5–1 × 10^6^Da), intermediate molecular weight (1–1,8 × 10^6^Da), and high molecular weight (6 × 10^6^ Da) (12). However, the hyaluronic acid used in the present study has 2.3 × 10^6^Da, according information of the company. There is controversy about the advantages of the different molecular weights of the hyaluronic acid when used *in vivo* (12). Theoretically, the hyaluronic acid used in the present study has effect for a few months due to its molecular weight, unlike longer-lasting effect products. On the other hand, because is a reticulated hyaluronic acid, repeated applications would not be necessary. There is evidence that cross-linking is responsible to extend the duration of intra-articular of hyaluronic acid (32).

Improvement in scores of orthopedic examination of the hip joints and lameness score in both groups, as well as positive percentage change of the PVF (%BW) in 71.43% hind limbs, but with no changes in the radiographic score, NA or goniometric assessment, suggested a positive effect of viscosupplementation to provide pain-relief. On the other hand, a total of 28.57% of the hind limbs had a negative percentage change of the PVF, suggesting a worse function of theses hind limbs (27) despite of the treatments. In a study in dogs with osteoarthritis related to hip dysplasia, lower pain scores and improved clinical signs were observed with a single intra-articular injection of hyaluronic acid (molecular weight 500–730 kDa) compared to intra-articular saline injection in combination with oral nutraceutical and carprofen (10). Also, in a study with dogs with arthritis in one joint (shoulder, elbow, carpus, stifle and tarsus) that were treated by two intra-articular injections of high molecular weight sodium hyaluronate (Hylartil−4.000.000), applied at 3 week interval, or carprofen orally, was found that at 6 weeks the sodium hyaluronate group was significantly better (58% fully recovered and 10% without improvement) compared anti-inflammatory group (8). In addition, in dogs with patellar luxation treated surgically that received sodium hyaluronate (molecular weight 500–730 730 kDa) injected intra-articularly at the time of the procedure, or at the time of the procedure and 1 week postoperative, had improved clinical scores in comparison to control group at the evaluation 4 weeks after surgery (9).

On the other hand, in studies with experimental induced cranial cruciate ligament rupture, no improvement was detected with the use of intra-articular hyaluronic acid (33, 34). In human patients there is also much controversy concerning intra-articular viscosupplementation, with studies showing positive results after administration (11, 35), while other studies have found no benefit (36). The great variety in preparations, number, and technique of applications and heterogeneity of osteoarthrosis cases may contribute for the different results (13, 37). These types of differences also occurs in clinical (8–10) and experimental studies in dogs (14, 33, 34, 37, 38).

Regarding to Group 2 (hyaluronic acid associated with ozone gas), the ozone concentration was 45 μg/mL. In human patients, concentrations from 20 to 30 μg/mL have shown a positive effect of the ozone therapy in the treatment of osteoarthritis, but the studies show lack of procedure standardization (18–20). In general, ozone alone has been administered 1–3 times per week, for 4–6 consecutive weeks or more (16–20). Since in the present study the gas was combined with hyaluronic acid, a single application was used.

The percent changes of the PVF (%BW) and VI (%BW × s) were statically significant in favor of the Group 2 compared with Group 1, which indicates a positive increase compared to baseline (27). In general, the PVF (largest force) and VI (area under the force-time curve) are decreased during lameness (27), suggesting that intra-articular ozone may have contributed in reducing the pain (14, 16, 19). However, should be considered that despite randomization, radiographic scores were higher in G1 than G2. On the other hand, the radiographic findings did not influenced the scores of orthopedic examination or lameness score. Thus, the absence of difference between two groups for other parameters had suggested that a single application of ozone was not able to avoid radiographic progression of osteoarthritis and improvement in hip extension and flexion. In a comparative study in human patients with knee osteoarthrosis, the group that received intra-articular injection of hyaluronic acid in combination with oxygen ozone showed better outcome than hyaluronic acid or ozone administered separately, but the applications were once a week for five consecutive weeks (39). Thus, further studies are necessary to clarify, including an ozone group, which may considered one of the limitations of the present study. Because intra-articular route in dogs generally requires sedation and/or anesthesia, one option would be rectal insufflation, as used in human patients with rheumatoid arthritis (40).

Another limitation of this study was the use of heterogeneous groups of dogs, which makes difficult the kinetic evaluation (41). In addition, the dogs were evaluated walking, because due the disease the dog may be unable to trot or have difficult to gait trial repetition (41), despite of trotting gait be considered more sensitive than walking gait to lameness detection (42). Thus, to avoid these influences future studies using dogs of the same breed and with the same hip scoring should be considered.

## Conclusion

In conclusion, the intra-articular viscosupplementation alone or associated with ozone gas allowed improvement of lameness scores and orthopedic examination score, but on Group 2 the association of ozone gas allowed better kinetic results.

## Data Availability Statement

The datasets generated for this study are available on request to the corresponding author.

## Ethics Statement

This study was approved by the Ethics Committee for the Use of Animals (no. 0101/2018—CEUA) of the School of Veterinary Medicine and Animal Science—São Paulo State University (UNESP), Botucatu, Brazil. Written informed consent was obtained from the owners for the participation of their animals in this study.

## Author Contributions

JS, SR, IS, and DM contributed to conception and design of the study. FM and MM performed ultrasound and analyzed the radiographs. RT was responsible for anesthesia. LC done the statistical analysis. JS and SR wrote the original draft of this manuscript. All authors contributed to manuscript revision, read, and approved the submitted version.

## Conflict of Interest

The authors declare that the research was conducted in the absence of any commercial or financial relationships that could be construed as a potential conflict of interest.

## Footnotes

^1^Hialurox; São Carlos, São Paulo, Brazil.

^2^Ozone & Life; São José dos Campos, São Paulo, Brazil.

^3^MyLab Alpha, Esaote® Monções, São Paulo, Brazil.

^4^GE Health, DR-F; Barueri, São Paulo, Brazil.

^5^ClearCanvas Workstation® Toronto, Canada.

^6^PVC Carci; Carci®, São Paulo, Brazil.

^7^Walkway High Resolution HRV4; Tekscan Inc. South Boston, MA, USA.

^8^Walkway 7.0 software; Tekscan Inc., South Boston, Massachusetts, USA.
